# Complete Genome Sequence of a Velogenic Newcastle Disease Virus Strain Isolated from a Clinically Healthy Exotic Parakeet (*Melopsittacus undulatus*) in Pakistan

**DOI:** 10.1128/genomeA.01581-16

**Published:** 2017-02-09

**Authors:** Abdul Wajid, Asma Basharat, Taseer Ahmed Khan, Muhammad Wasim, Shafqat Fatima Rehmani

**Affiliations:** a Institute of Biochemistry and Biotechnology (IBBt), University of Veterinary and Animal Sciences, Lahore, Pakistan; b Quality Operations Laboratory (QOL), University of Veterinary and Animal Sciences, Lahore, Pakistan; c Department of Physiology, University of Karachi, Karachi, Pakistan

## Abstract

The complete genome sequence of a virulent Newcastle disease virus (vNDV) strain isolated from an exotic parakeet (*Melopsittacus undulatus*) is described here. The virulent strain parakeet/Pak/R-Pindi/SFR-16/2016 was isolated from a bird reared as a pet in the province of Punjab in the northern region of Pakistan in 2016. Phylogenetic analysis classified the isolate as a member of NDV class II, subgenotype VIIi, in genotype VII.

## GENOME ANNOUNCEMENT

Newcastle disease (ND) is the one of the most economically important viral diseases affecting many species of birds. Newcastle disease is a devastating disease of poultry worldwide due to high mortality in commercial young chickens (1). Newcastle disease virus (NDV) is classified as an avian paramyxovirus type 1 (AMPV-1), belonging to the genus *Avulavirus*, family *Paramyxoviridae*, order *Mononegavirales* (2). NDV is an enveloped virus having a negative-sense, nonsegmented, and single-stranded RNA, with a genome length of 15.2 kb (3, 4). The NDV genome encodes six transcriptional units, nucleocapsid protein (NP), phosphoprotein (P), matrix protein (M), fusion protein (F), hemagglutinin-neuraminidase protein (HN), and large protein (L), with an additional V protein that is expressed by RNA editing of P mRNA (5).

An NDV isolate designated parakeet/Pak/R-Pindi/SFR-16/2016 (SFR-16) was identified and classified as a member of the newly emerging subgenotype VIIi in poultry production facilities and pet birds in Pakistan. Viruses classified in this subgenotype are widely spread through Asia, the Middle East, eastern European countries, like Turkey, Georgia, Bulgaria, and recently in Indian peafowl causing outbreaks of virulent NDV (vNDV) with high mortality in poultry flocks, suggesting the existence of a fifth panzootic (6–8). Nonsignificant changes in the complete genome sequences of ND viruses recently reported in chickens, ducks and parakeets in Pakistan revealed apparent spill-over of the viruses among them (7).

Oropharyngeal and cloacal swabs, along with blood samples, were collected from healthy parakeets and inoculated into 9- to 10-day-old (NDV-specific-antibody-free) chicken eggs. A hemagglutination assay (HA) was performed, and NDV was suspected by a positive HA test result (9). The positive HA allantoic fluid was used for viral RNA extraction using TRIzol LS (Invitrogen, USA), according to the manufacturer’s protocol. The first-strand cDNA (cDNA) was synthesized using the Thermo Scientific reverse transcriptase PCR (RT-PCR) kit using random hexamer (Thermo Scientific, USA), according to the manufacturer’s recommendations. The complete genome was sequenced as described previously (3), and the BioEdit software was used for the sequence analysis (10).

Sequence analysis showed that the complete genome length of the SFR-16 isolate was 15,192 nucleotides (nt). The SFR-16 strain has the highest homology (>99%) to the sequences of previously isolated ND viruses from duck (AW-123) and chicken (SFR-611) (GenBank accession numbers KU845252 and KM670337, respectively). The amino acid sequence identities of the NP, P, M, HN, F, and L proteins between SFR-16 and AW-123 strains are 99.38%, 100%, 99.17%, 99.82%, 99.27%, and 100%, respectively. SFR-16 was classified as velogenic NDV, with an intracerebral pathogenicity index (ICPI) of 1.70 in day-old specific-NDV antibody-free chicks; the result is consistent with the polybasic amino acid motif and a polyethylene at position 117 (^112^RRQKR↓F^117^) at the fusion protein cleavage site, which is typical for virulent NDV isolates (9). The current scenario of the highly related viral transmission in various pet birds in Pakistan highlights the importance of an active surveillance program.

### Accession number(s).

The complete genome sequence of NDV strain parakeet/Pak/R-Pindi/SFR-16/2016 has been deposited in GenBank under the accession number KX791183.
